# Weight Trajectories Among Youths Following Residential Relocation

**DOI:** 10.1001/jamanetworkopen.2025.44164

**Published:** 2025-11-18

**Authors:** Apolline Saucy, Sarah Warkentin, Carles Milà, Fabián Coloma, Zhebin Yu, Jeroen de Bont, Anna Bergström, Jolanda M.A. Boer, Payam Dadvand, Kees de Hoogh, Ulrike Gehring, Jana Klánová, Ondřej Mikeš, Erik Melén, Mark Nieuwenhuijsen, Youchen Shen, Daniel Szabó, Roel Vermeulen, Jelle Vlaanderen, Judith M. Vonk, Cathryn Tonne

**Affiliations:** 1 ISGlobal, Barcelona, Spain; 2 Universitat Pompeu Fabra, Barcelona, Spain; 3 Spanish Consortium for Research on Epidemiology and Public Health (CIBERESP), Madrid, Spain; 4 Institute of Environmental Medicine, Karolinska Institutet, Stockholm, Sweden; 5 Centre for Occupational and Environmental Medicine, Region Stockholm, Stockholm, Sweden; 6 National Institute for Public Health and the Environment, Bilthoven, the Netherlands; 7 Swiss Tropical and Public Health Institute, Basel, Switzerland; 8 University of Basel, Basel, Switzerland; 9 Institute for Risk Assessment Sciences, Utrecht University, Utrecht, the Netherlands; 10 RECETOX, Faculty of Science, Masaryk University, Brno, Czech Republic; 11 Department of Clinical Science and Education, Södersjukhuset, Karolinska Institutet, Stockholm, Sweden; 12 Department of Epidemiology & Groningen Research Institute for Asthma and COPD, University of Groningen, University Medical Center Groningen, Groningen, the Netherlands

## Abstract

**Importance:**

Overweight and obesity affect millions of children and adolescents worldwide, and its prevalence is increasing.

**Objective:**

To investigate the associations of changes in the surrounding residential environment following relocation on childhood body mass index (BMI), focusing on 3 external exposome domains: air pollution, the built environment, and socioeconomic disadvantage.

**Design, Setting, and Participants:**

This longitudinal cohort study used harmonized data from birth cohorts from the Netherlands (PIAMA), Sweden (BAMSE), and the Czech Republic (ELSPAC-CZ) participating in the EXPANSE (Exposome Powered Tools for Healthy Living in Urban Settings) project, with birth dates ranging between 1991 and 1997. Participants were youths aged 2 to 24 years who had experienced residential relocation during their follow-up. Analysis focused on within-individual changes resulting from relocation. *k* Means clustering characterized multiple exposures from the 3 external exposome domains. Fixed-effects linear models estimated associations of exposome changes with changes in age- and sex-standardized body mass index (z-BMI), adjusted for relevant covariates. This study was conducted between July 2023 and January 2025.

**Exposures:**

Changes in 3 external exposome domains: (1) ambient air pollution from high-resolution surfaces; (2) the built environment, including green, blue, and gray spaces and light at night; and (3) area-level socioeconomic disadvantage indicators. Domain-specific exposome profiles were characterized as low-, medium-, and high-hazard environments.

**Main Outcome and Measures:**

Changes in z-BMI.

**Results:**

The study included 4359 participants (1467 from PIAMA, 1778 from BAMSE, and 1114 from ELSPAC-CZ). A total of 2215 (50.8%) were male. The mean (SD) age at inclusion was 3.0 (1.1) years, and mean (SD) age at moving was 7.7 (4.3) years. Parental education varied across cohorts. Mean (SD) z-BMI was 0.2 (1.1), 0.4 (1.0), and 0.1 (1.2) at baseline and 0.0 (1.0), 0.3 (1.0), and 0.1 (1.1) after moving in PIAMA, BAMSE, and ELSPAC-CZ, respectively. Moving to higher-hazard environments (more polluted, more gray space) was associated with increases in z-BMI for all domains in PIAMA; significant associations were also seen for some domains and exposures in BAMSE and ELSPAC-CZ. Specifically, an association between moving to a more built environment and increase in z-BMI was consistent across cohorts: an IQR increase in gray spaces was associated with increases of 0.04 (95% CI, 0.01-0.06) units and 0.05 (95% CI, 0.01-0.09) units in z-BMI in BAMSE and PIAMA, respectively. An IQR increase in air pollution hazard was associated with increases of 0.07 (95% CI, 0.02-0.12) units and 0.07 (95% CI, 0.01-0.14) units in z-BMI for nitrogen dioxide (NO_2_) and fine particulate matter (PM_2.5_), respectively, in PIAMA. Presence of effect modification by parental education and age at moving varied across cohorts.

**Conclusions and Relevance:**

In this multicountry cohort study of 4359 youths in the Netherlands, Sweden, and the Czech Republic, moving to greener, less urbanized environments was associated with healthy childhood BMI trajectories. Heterogeneity across cohorts highlighted the context-specific influence of external exposome domains on childhood weight.

## Key Points

**Question:**

Is moving to a different environment associated with body mass index (BMI) trajectories in young people through changes in the external exposome?

**Findings:**

In this cohort study of 4359 children and young adults (aged 2-24 years) in the Netherlands, Sweden, and the Czech Republic with more than 30 000 age- and sex-standardized BMI (z-BMI) observations, moving to areas with higher environmental hazards (ie, more air pollution or less green space) was associated with increases in z-BMI, particularly in the Dutch cohort, with similar associations seen with gray space in the Swedish cohort; the Czech cohort showed no clear associations.

**Meaning:**

These findings suggest that greener, less polluted environments may help prevent unhealthy BMI trajectories in children and adolescents, with potential benefits differing across exposome domains and cohorts.

## Teaser

This cohort study investigates the associations of changes in the surrounding residential environment—specifically related to air pollution, the built environment, and socioeconomic disadvantage—with childhood body mass index trajectories.

## Introduction

Overweight and obesity are among the leading causes of mortality and morbidity globally, contributing to 4.72 million deaths (8% of all global deaths) and 148 million disability-adjusted life years in 2017.^1^ More than 340 million children and adolescents aged 5 to 19 years have overweight or obesity, and rates are increasing.^2^ The global prevalence of obesity in school-aged children quadrupled between 1990 and 2022, especially in low-income countries, mostly due to the increase in sedentary lifestyles and high-calorie food intake.^3^ In Europe, despite several public health policies, the prevalence of childhood overweight remains high and is still increasing in several countries, especially in Northern European countries.^4^ The complex, multifactorial conditions of overweight and obesity are, however, largely preventable.^5^

The living environment may play an important role in maintaining healthy weight.^6^ Early life obesogenic exposures seem particularly relevant in shaping later body mass index (BMI) trajectories, but the timing and mechanisms of these effects remain unclear.^7,8^ Early-life environmental exposures, including in utero and during childhood, may influence later BMI through multiple pathways, such as endocrine and inflammatory processes,^9^ or by shaping behaviors like physical activity and diet.^10^ For example, environmental conditions, including air pollution,^9^ environmental noise,^11^ green spaces,^12^ and the built environment,^13^ have been associated with changes in children’s growth and weight.^9,13^ de Bont et al^14^ found an association of in utero exposure to air pollution, green space, and the built environment with early-life BMI trajectories, while Wallas et al^11^ reported that exposure to environmental road traffic noise in utero and during childhood was associated with a higher BMI in school age and adolescence, but not at earlier ages. Tackling childhood overweight is of particular interest because (1) many health behaviors are learned during early life, making it an important time window for public health interventions^15^; and (2) early interventions can prevent both immediate (eg, poor respiratory and mental health^5^) and long-term (eg, high blood pressure, diabetes, cancer) outcomes of having overweight.

Previous studies have identified a need for more research on the obesogenic potential of the built environment, especially across the life course,^8^ using causal frameworks such as quasi-experimental studies,^16^ and evaluating the potential of changes in the living environment, especially at a young age.^17,18^ In addition, as most studies focusing on childhood BMI have evaluated the impact of single or few environmental factors, there is a need for studies simultaneously evaluating coexisting environmental and socioeconomic factors to both provide a more realistic picture and identify the most impactful factors. The exposome framework is a useful tool to address these gaps by focusing on the totality of environmental exposures experienced over the life course^19^ and offers a unique opportunity to leverage large individual and environmental datasets for residential relocation studies.^20^ Residential relocation studies are valuable in exposome research as they enable the investigation of causal relationships by analyzing within-individual changes in multiple environmental exposures and health outcomes, while addressing confounding factors such as residential self-selection and socioeconomic differences.^20^

We adopted an exposome approach to evaluate the association of residential relocation trajectories in childhood with early adulthood BMI trajectories, using a quasi-experimental study design. Specifically, we used harmonized data from 3 European birth cohorts to (1) characterize changes in 3 domains of the external exposome (air pollution, the built environment, and area-level socioeconomic disadvantage) resulting from residential relocation; and (2) assess the association between these changes in the external exposome and standardized BMI trajectories.

## Methods

This cohort study is based on secondary analysis of preexisting cohort data. Each cohort obtained approval from its local ethics committee, and all data were provided by the cohorts in anonymized form, preventing identification of individual participants. This work was conducted in accordance with the Strengthening the Reporting of Observational Studies in Epidemiology (STROBE) reporting guideline.^21^

### Study Population

We included data from 3 birth cohorts participating in the EXPANSE project (Exposome Powered Tools for Healthy Living in Urban Settings): PIAMA (Prevention and Incidence of Asthma and Mite Allergy), BAMSE (Children, Allergy, Milieu, Stockholm, Epidemiology) and Czech ELSPAC (European Longitudinal Study of Pregnancy and Childhood). PIAMA is an ongoing population-based birth cohort including 3963 children born between 1996 and 1997 in the Netherlands.^22^ BAMSE is an ongoing longitudinal, population-based prospective birth cohort including 4089 children born between 1994 and 1996 in Stockholm, Sweden.^23^ The Czech ELSPAC study (ELSPAC-CZ) includes children born in the Brno and Znojmo regions (South Moravia, Czech Republic) between 1991 and 1992 and was set up as a part of the ELSPAC study.^24,25^ The full baseline dataset included 7589 children, with data collected from medical records during the first study wave. Although only 5151 mothers completed self-reported questionnaires and were considered cohort participants, for the purposes of this study, we used the full baseline dataset. For all 3 cohorts, full residential histories (lists of geocoded consecutive addresses with exact moving dates) were derived from regular questionnaires and/or population registries.

### Outcome Data and Covariates

Age- and sex-standardized BMI values (z-BMI) were calculated for all cohorts following the World Health Organization (WHO) growth reference charts^26,27^ using BMI measurements (weight in kilograms divided by height in meters squared) collected from different sources within the cohorts (Table 1). For participants aged 18 years or older (up to 24 years), the WHO age-18 formula was applied. In PIAMA, BMIs with exact measurement dates were available from health questionnaires implemented in 13 waves and clinical examinations in 4 waves. In BAMSE, body weight and height measurements were collected during regular physical examinations in 4 waves (all children) and retrospectively linked to data from school medical visits and health records for a subsample of the children. BMI data derived from school medical visits were available for predefined ages and did not include the exact measurement date (±6 months for data until 5 years of age and −6 to +11 months from 7 years onwards) and represented 64% of all collected BMI observations in BAMSE.^11^ In ELSPAC-CZ, BMI data were derived from continuous medical visits with exact measurement dates. Individual covariate data included child age, sex (male or female), nationality (has country’s nationality, yes or no) and highest parental education (low, medium, or high). Additionally, we calculated age and time lived at the current address for each z-BMI value and exact age at residential relocation, and we indicated whether BMI values were measured or self-reported.

**Table 1.** Summary Table of Study Population and Cohorts Characteristics

Characteristics	BAMSE	ELSPAC-CZ	PIAMA
BMI data source	Physical examination at ages 4, 8, 16, 24 y. School medical visits at ages 2, 3, 4, 5, 7, 10, 12 y.	Medical visits (continuous) at ages 2 to 19 y.	Self-reported: yearly from ages 2-8 y, then 11, 14, and 17 y, and self-reported at 20 y. Physical examination at ages 4, 8, 12, and 16.
Individuals, No.	1778	1114	1467
BMI observations, No.	11 958	7774	11 331
Sex at birth, No. (%)			
Female	871 (49.0)	541 (48.6)	731 (49.8)
Male	907 (51.0)	573 (51.4)	735 (50.1)
Has country’s nationality, No. (%)			
Yes	1435 (84.1)	NA	1312 (90.9)
No	272 (15.9)	NA	131 (9.1)
Highest parental education, No. (%)			
Low	37 (2.1)	24 (3.6)	151 (10.5)
Medium	799 (45.0)	412 (62.6)	503 (34.9)
High	941 (53.0)	217 (33.0)	787 (54.6)
Age at inclusion, mean (SD), y	3.0 (1.6)	3.1 (0.5)	2.9 (0.7)
Age at moving, mean (SD), y	8.3 (5.4)	7.4 (2.7)	6.8 (3.5)
Before moving			
Years spent at current address, mean (SD)	4.7 (3.1)	5.1 (2.8)	4.7 (2.5)
z-BMI, mean (SD)	0.4 (1.0)	0.1 (1.2)	0.2 (1.1)
After moving			
Years spent at current address, mean (SD)	4.7 (4.2)	5.6 (3.4)	5.2 (4.1)
z-BMI, mean (SD)	0.3 (1.0)	0.1 (1.1)	0.0 (1.0)
Temporal extent	1996-2020	1993-2011	1998-2017

Abbreviations: BAMSE, Children, Allergy, Milieu, Stockholm, Epidemiology study; BMI, body mass index (calculated as weight in kilograms divided by height in meters squared); ELSPAC-CZ, Czech European Longitudinal Study of Pregnancy and Childhood; NA, not applicable; PIAMA, Prevention and Incidence of Asthma and Mite Allergy; z-BMI, age- and sex-standardized body mass index.

### Study Design

We conducted a movers-only quasi-experimental cohort study. Because our study aims to understand the impact of exposome change due to relocation, our analyses focused on within-individual exposome variation among movers. All children aged 2 years or older who relocated during the follow-up and with available pre- and post-moving z-BMI data were included in the analysis due to difficulty interpreting healthy BMI before 2 years.^28^ Follow-up started at the first valid z-BMI observation after 2 years of age and continued until the end of the cohort’s observation period or earlier if the participant was lost to follow-up or relocated a second time. z-BMI observations were included only if they occurred while the child resided at an address with available geocodes and if the child had resided at the same address for at least 3 months before the observation, since substantial changes in z-BMI are unlikely to occur over a shorter period following relocation. z-BMI observations were excluded for having less than 3 months since moved, for occurring after a second move, and because of a missing address. We excluded children who moved frequently (at least 10 previous registered addresses) because this has been associated with adverse early life experiences and health outcomes.^29^ A flow diagram with detailed description of the data preparation and exclusions is provided in the eMethods in Supplement 1.

### Exposome Assessment

Individual exposure at the home address was estimated considering 3 domains of the external exposome: (1) air pollution (including, nitrogen dioxide [NO_2_], ozone [O_3_] and particulate matter <2.5 µg/m^3^ [PM_2.5_] and <10 µg/m^3^ [PM_10_]); (2) the built environment (including green [vegetation indices], gray [imperviousness], and blue [water bodies] surfaces within 300- and 500-meter buffers of home address, and light-at-night); and (3) area-level socioeconomic disadvantage (including, socioeconomic position index and unemployment rates). For the air pollution and built environment domains, unified exposure for the European region was available from EXPANSE.^30^ For the socioeconomic disadvantage domain, for which no harmonized data were available at the European level, we collected the best available country-specific area-level socioeconomic variables in each cohort. A detailed overview of the exposome variables, including spatial resolution and temporal extent, is available in Table 2 and in the eMethods in Supplement 1.^31,32^ In brief, exposome data were available for 1 or several years, ranging from 2000 to 2020, and were extracted at pre- and post-move addresses for all included participants. Whenever exposure variables were available for several years, we selected the year closest to the date of moving. z-BMI and exposome data do not always temporally coincide due to the long duration of the cohorts—with z-BMI data from 1993 onward—and the availability of exposome models. However, spatial differences between old and new addresses are expected to dominate over small long-term trends, which, together with restriction to the first move, minimizes potential exposure misclassification.

**Table 2.** List of EXPANSE Exposures Available at the Time of Analysis

Domain and exposures^a^	Spatial resolution	Temporal extent
**Air pollution**		
NO_2_, PM_2.5_, PM_10_, O_3_	100 × 100 m	Annual (2000-2019)
**Built environment**		
Green surface (NDVI and MSAVI, 300 m and 500 m buffers)	250 × 250 m	2000-2020 (Annual averages every 5 y)
Distance to nearest green space from CORINE	100 × 100 m	2000-2018 (Annual averages every 6 y)
Impervious surface (500 m buffer)	100 × 100 m	2006-2018 (Annual averages every 3 y)
Light at night (500 m buffer)	100 × 100 m	2000-2020 (Annual averages every 5 y)
Green SD (300 m and 500 m buffers for NDVI and MSAVI surfaces)	250 × 250 m	2000-2020 (Annual averages every 5 y)
Distance to blue and freshwater	100 × 100 m	2013 (Annual average)
**Socioeconomic disadvantage^b^**		
Area-level socioeconomic position	Cohort-specific	Cohort-specific

Abbreviations: BAMSE, Children, Allergy, Millieu, Stockholm, Epidemiology study; CORINE, Copernicus Land Monitoring Service Land Cover; ELSPAC-CZ, Czech European Longitudinal Study of Pregnancy and Childhood; EXPANSE, Exposome Powered Tools for Healthy Living in Urban Settings; MSAVI, Modified Soil Adjusted Vegetation Index; NDVI, Normalized Difference Vegetation Index; NO_2_, nitrogen dioxide; O_3_, ozone; PIAMA, Prevention and Incidence of Asthma and Mite Allergy; PM_10_, particulate matter with diameter <10 µg/m^3^; PM_2.5_, particulate matter with diameter <2.5 µg/m^3^.

^a^ The year of exposure was selected for each individual to be closest to the date of the first residential relocation.

^b^ Exposures of the socioeconomic environment were collected for each cohort separately according to availability. For PIAMA, we used the percentage of low-income persons at the neighborhood level, defined as “part of a municipality dominated by a given type of land use or buildings. For instance: industrial area, residential area with high-rise or low-rise buildings.”^31^ For BAMSE, the socioeconomic data were mean income at the neighborhood level from the Swedish National Statistical Office (Statistics Sweden, 2000). In Sweden, neighborhoods are defined as Small Area Market Statistics (SAMS), which refer to “the smallest areal units in a system of geographical coordinates areas in Stockholm and 9281 SAMS areas in the rest of Sweden.”^32^ For ELSPAC-CZ, we used the percentage of unemployment from the economically active population at census level (>300 units for the city of Brno).

We then characterized the domain-specific external exposome at home addresses into increasing hazard groups (low, medium, and high) using *k* means clustering based on the Hartigan and Wong algorithm^33^ as described previously.^34^ To ensure comparability within domains, all exposures were rescaled separately for each cohort using the cohort-specific IQR distribution. For the socioeconomic disadvantage domain, where a single variable was available, hazard groups were assigned based on the tertile distribution of country-specific area-level socioeconomic indicators. Changes in exposome characteristics reflect geographical differences between consecutive home addresses at a given time to avoid potential confounding by time trends.^34^

### Statistical Analyses

Analyses were conducted between July 2023 and January 2025. We estimated the association between change in each exposome domain due to residential relocation and change in z-BMI using fixed-effects linear models for panel data, with exposome groups as a categorical variable. Fixed-effects models, similar to difference-in-differences models, focus on within-individual associations^35,36^ and thus are able to estimate the associations of changes in hazard group membership due to relocation with subsequent changes in z-BMI. Whereas difference-in-differences models take advantage of an intervention outside the control of the individual and are well designed for population-based analyses, fixed-effects models leverage individual-level data with repeated observations. This is a movers-only approach, wherein each person serves as their own control. Fixed-effects models are particularly useful for relocation studies, as they can accommodate longitudinal study designs with repeated measurements and different timings of the intervention, as well as avoid the risk of confounding by time-invariant confounders (eg, sex at birth, parental education, and other family characteristics) between individuals.^35,37^ Relevant time-invariant characteristics were, however, considered important effect modifiers and were included in sensitivity analyses described below.

To estimate the crude association between changes in individual exposures and changes in z-BMI, we fitted single-exposure fixed-effects linear models and reported estimates for an (exposure-specific) IQR increase in exposure. We then estimated the association between changes in each exposome domain and changes in z-BMI (single-domain models with separate models for each domain of the external exposome) and fitted a multiple exposure–adjusted model including all 3 domains simultaneously using the following equation for the final model, with fixed effects at the individual level for *i* = 1,…,N and time *t* = 1, …, T:

z-BMI_it_ = α_i_ + β_1_ × AP_it_ + β_2_ × BS_it_ + β_3_ × SEP_it_ + β_4_ × moved_it_ + β_5_ × age_it_ + β_6_ × age_it_^2^ + β_7_ × self-report + ε_it_,

where α_i_ are child-specific intercepts corresponding to a fixed effects estimation, AP is the categorical air pollution exposome domain, BS is the built environment, and SEP is the socioeconomic disadvantage domain. Moved is the dummy variable for relocation status, age is the centered child’s age in months at time *t*, self-report is the dummy variable for measurement type (self-reported BMI), and ε is the error term.

To account for time-varying characteristics, all models included a dummy variable for relocation status (before and after) as well as measurement type (eg, self-reported, medical visit) and centered age including a squared term to account for potential time trends not accounted for by the z-BMI standardization. We explored potential effect modifications by sociodemographic characteristics by performing stratified analyses by sex, age at moving, and parental education in the final model. In sensitivity analyses, we tested the validity of the exposure time window by excluding z-BMI measurements within 6 months and 1 year of the relocation. All analyses were performed separately for each cohort using R, version 4.0.5 (plm package) (R Foundation).

## Results

Our initial dataset included 148 717 repeated z-BMI observations from 15 239 children and young adults (3963 in PIAMA, 4089 in BAMSE, and 7589 in ELSPAC-CZ). After applying exclusion criteria (outliers, missing addresses, age <2 years, residence <3 months, multiple relocations, and restricting to movers only), the analytical sample constituted 11 331 observations from 1467 individuals in PIAMA, 11 958 observations from 1778 individuals in BAMSE, and 7774 observations from 1114 individuals in ELSPAC-CZ (eMethods in Supplement 1). A total of 2215 (50.8%) were male. The mean (SD) age at inclusion was 3.0 (1.1) years, and mean (SD) age at moving was 7.7 (4.3) years. In PIAMA, 731 individuals (49.8%) were female and 735 (50.1%) male; mean (SD) age was 2.9 (0.7) years at inclusion and 6.8 (3.5) years at moving; 787 (54.6%) had high parental education; and mean (SD) baseline z-BMI was 0.2 (1.1). In BAMSE, 871 individuals (49.0%) were female and 908 (51.0%) male; mean (SD) age was 3.0 (1.6) years at inclusion and 8.3 (5.4) years at moving; 941 (53.0%) had high parental education; and mean (SD) baseline z-BMI was 0.4 (1.0). In ELSPAC-CZ, 541 individuals (48.6%) were female and 573 (51.4%) male; mean (SD) age was 3.1 (0.5) years at inclusion and 7.4 (2.7) years at moving; 217 (33.0%) had high parental education; and mean (SD) baseline z-BMI was 0.1 (1.2). Individual and study characteristics are described in Table 1. BAMSE participants had the lowest exposure to particulate matter and impervious surfaces and the highest greenness (eFigure 1 in Supplement 1). The highest PM_2.5_ exposure was observed in ELSPAC-CZ participants. PIAMA and ELSPAC-CZ participants had comparable NO_2_ and PM_10_ exposures (eFigure 1 in Supplement 1). Changes in individual exposures upon relocation were comparable across cohorts (eFigure 2 in Supplement 1).

**Figure 1.**
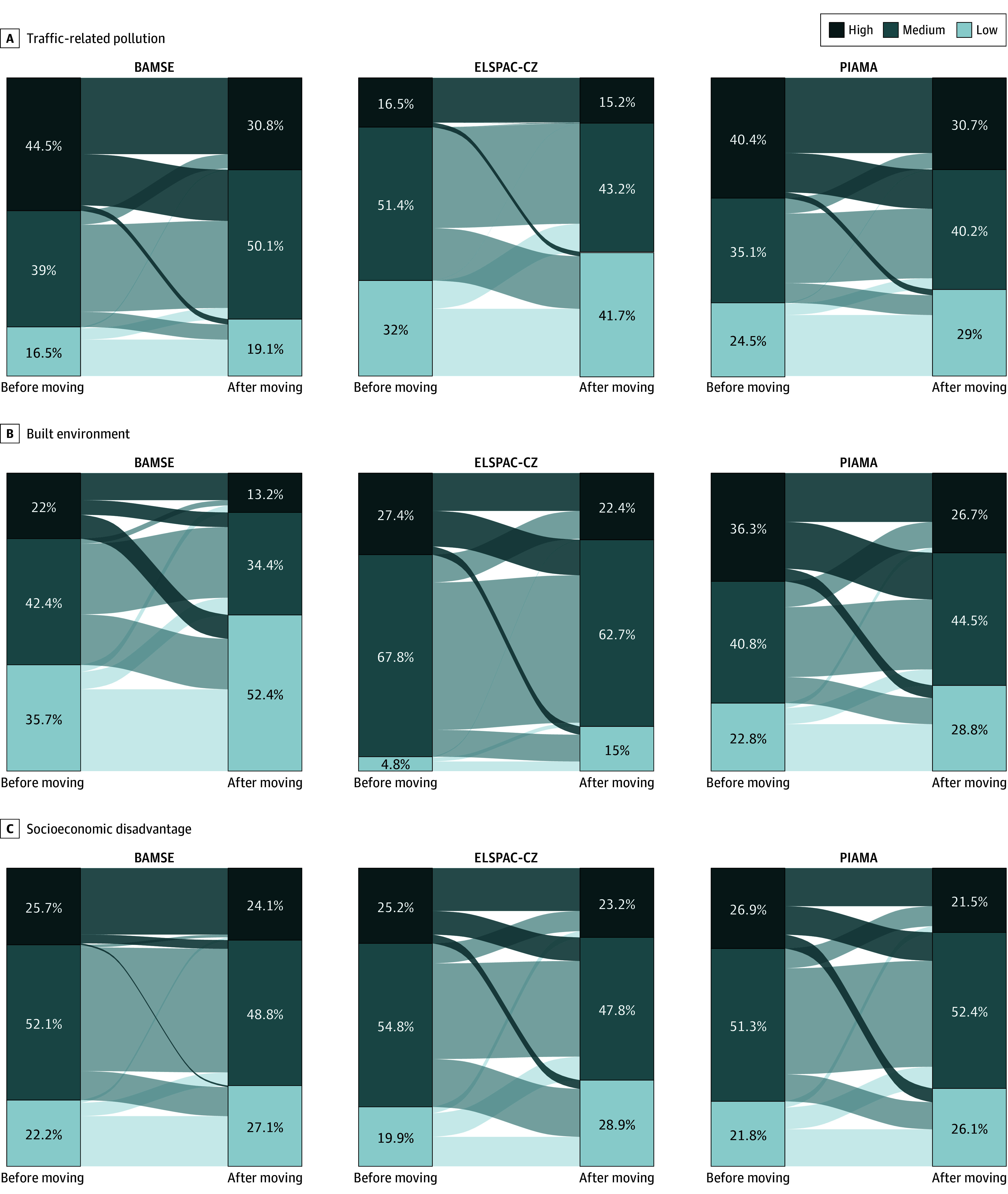
Changes in Cluster Levels for 3 Domains of the External Exposome Upon Moving Lower-hazard cluster levels represent lower levels of air pollution, built environment, and socioeconomic disadvantage. Numbers indicate the percentage of study participants in the different clusters before and after moving. Note that clusters were built separately for each cohort, and cluster distributions at given times cannot be compared across cohorts. BAMSE indicates the Children, Allergy, Milieu, Stockholm, Epidemiology study; ELSPAC-CZ, Czech ELSPAC (European Longitudinal Study of Pregnancy and Childhood); and PIAMA, Prevention and Incidence of Asthma and Mite Allergy.

The high air pollution cluster (high hazard) included residences with higher concentrations of PM_2.5_ and PM_10_ and lower O_3_ for all cohorts and higher NO_2_ for PIAMA and BAMSE (eFigure 3 in Supplement 1). ELSPAC-CZ had different exposure correlation structures compared to the two Northern European cohorts; the highest NO_2_ exposures fell within the medium hazard cluster. Regarding the built environment domain, the high hazard cluster included areas with the highest amount of impervious surface, light at night, and distance to green space and the lowest values of the various greenness indicators in all 3 cohorts (eFigure 3 in Supplement 1). Pearson correlations across exposures and exposome clusters are displayed in eFigures 4 to 7 in Supplement 1. Across all cohorts and exposome domains, relocation often resulted in changes within the same hazard cluster (eg, low to low or high to high). Relocating to healthier clusters (more green, less polluted, and higher SEP) was as common as relocating to more hazardous clusters. Changes in socioeconomic disadvantage hazard groups were limited in BAMSE (Figure 1; eFigure 8 in Supplement 1).

**Figure 2.**
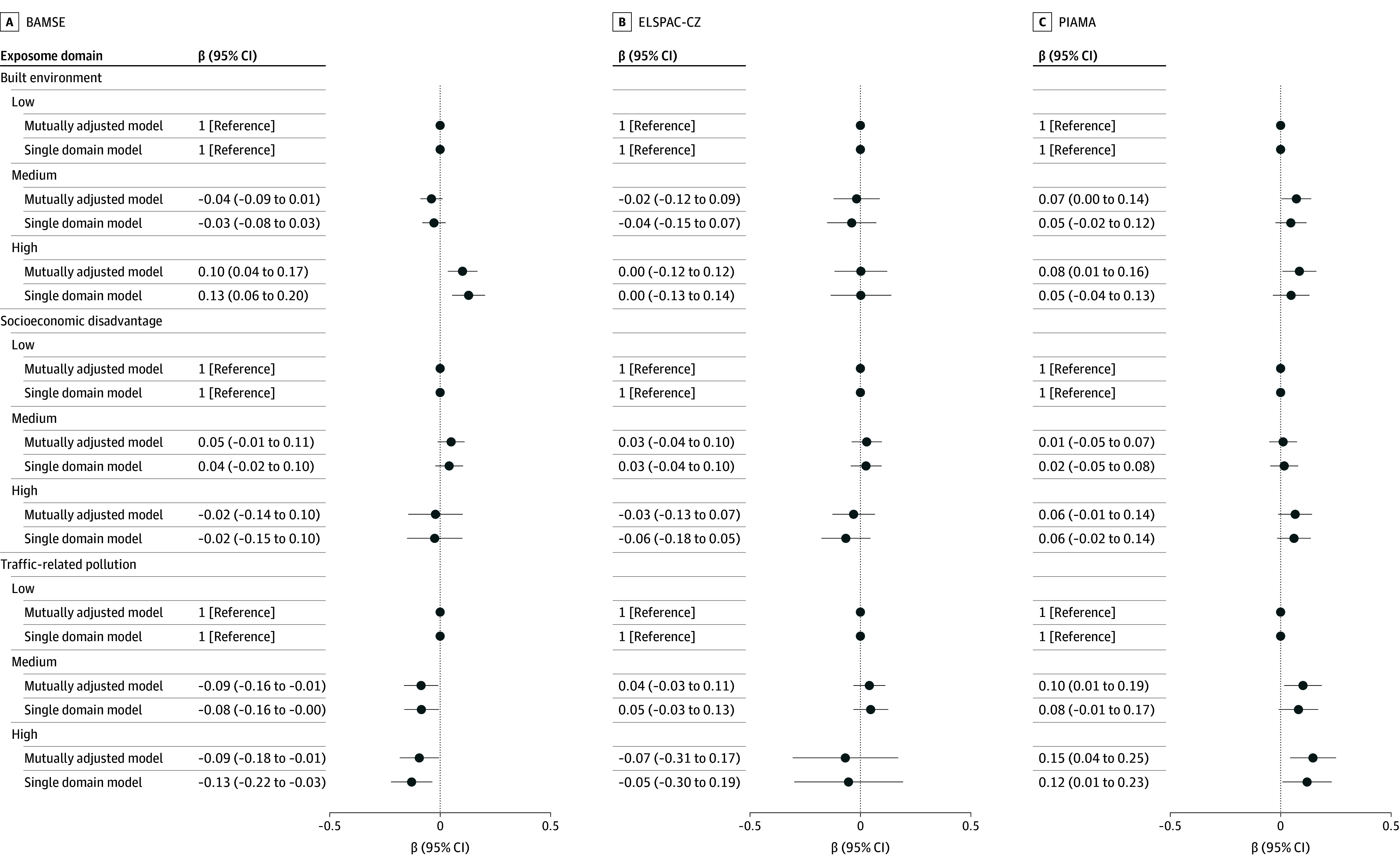
Association of Changes in Domain-Specific Exposome Cluster Groups With Changes in Age- and Sex-Standardized Body Mass Index (z-BMI) in the 3 Cohorts Coefficient estimates are displayed with 95% CIs. The figure displays changes in z-BMI associated with moving from the low to the medium cluster level and from the low to the high cluster level. BAMSE indicates the Children, Allergy, Milieu, Stockholm, Epidemiology study; ELSPAC-CZ, Czech ELSPAC (European Longitudinal Study of Pregnancy and Childhood); and PIAMA, Prevention and Incidence of Asthma and Mite Allergy.

Table 3 presents results from the single-exposure fixed effects models. Moving to areas with higher air pollution concentrations was associated with an increase in post-move z-BMI in PIAMA. Specifically, an IQR increase in NO_2_ was associated with an increase of 0.07 (95% CI, 0.02-0.12) units, and an IQR increase in PM_2.5_ was associated with an increase of 0.07 (95% CI, 0.01-0.14) units in z-BMI. Relocating to areas with more impervious surfaces and less greenness was associated with increases in post-move z-BMI in both PIAMA (0.05 [95% CI, 0.01-0.09] units) and BAMSE (0.04 [95% CI, 0.01-0.06] units). In ELSPAC-CZ, relocating to areas with more distance to green space was associated with an increase in post-move z-BMI (an IQR increase in 0.05 z-BMI increase per IQR; 95% CI, 0.01-0.08). Results from the domain-specific exposome cluster models are presented in Figure 2. We found consistent associations of relocating to higher hazard clusters with an increase in post-move z-BMI for all exposome domains (air pollution, built environment, and socioeconomic disadvantage) in PIAMA. The same findings were present in BAMSE for the built environment only, and the opposite association for the air pollution domain. Sensitivity analyses suggest that this last association was mostly driven by the subgroup of younger boys (aged ≤7 years) with high parental education (eTable in Supplement 1). Moving to higher hazard clusters for the built environment was associated with consistent increases in post-move z-BMI for children with lowest parental education in ELSPAC-CZ, but not in the main models. Full results from the sensitivity analyses are presented in the eTable in Supplement 1. Overall, patterns of effect modification varied across cohorts. There was no consistent evidence of effect modification by sex across cohorts or domains. The coefficient for changes in the built environment and post-move z-BMI increase was largest for the lowest parental education group in PIAMA and ELSPAC-CZ and for the highest parental education group in BAMSE. In PIAMA, the coefficient was largest in younger children (aged ≤7 years) for the air pollution domain and in older children (>7 years) for the socioeconomic disadvantage domain; there was no effect modification by age at moving for the built environment. The single-domain and multiple-domain (final) models produced similar results compared with the single-domain models, suggesting minimal mutual confounding in the association between the 3 external exposome domains and z-BMI changes (Figure 2).

**Table 3.** Association Between Changes in Individual Exposures Upon Moving and Changes in Age- and Sex-Standardized Body Mass Index in the 3 Cohorts

Exposure	β (95% CI)^a^		
	BAMSE (n = 1178)	ELSPAC-CZ (n = 1114)	PIAMA (n = 1467)
Air pollution, µg/m^3^			
NO_2_	0.01 (−0.02 to 0.05)	0.02 (−0.03 to 0.06)	0.07 (0.02 to 0.12)
O_3_	0.01 (−0.03 to 0.06)	−0.02 (−0.07 to 0.03)	0.00 (−0.08 to 0.09)
PM_10_	−0.03 (−0.07 to 0.00)	0.02 (−0.02 to 0.06)	0.06 (−0.03 to 0.15)
PM_2.5_	−0.05 (−0.10 to 0.00)	0.02 (−0.03 to 0.07)	0.07 (0.01 to 0.14)
Built environment			
Impervious surface, %	0.04 (0.01 to 0.06)	0.00 (−0.03 to 0.04)	0.05 (0.01 to 0.09)
Light at night, RAD	−0.01 (−0.01 to 0.00)	0.00 (−0.02 to 0.02)	0.00 (−0.04 to 0.03)
MSAVI, mean, 300 m	−0.04 (−0.07 to −0.01)	0.02 (−0.02 to 0.05)	−0.04 (−0.07 to 0.00)
MSAVI, mean, 500 m	−0.04 (−0.07 to −0.01)	0.02 (−0.02 to 0.06)	−0.03 (−0.07 to 0.01)
MSAVI, SD, 500 m	−0.01 (−0.04 to 0.02)	−0.02 (−0.07 to 0.03)	−0.01 (−0.04 to 0.02)
NDVI, mean, 300 m	−0.04 (−0.07 to −0.01)	0.01 (−0.02 to 0.05)	−0.04 (−0.07 to 0.00)
NDVI, mean, 500 m	−0.04 (−0.07 to −0.01)	0.01 (−0.03 to 0.05)	−0.03 (−0.07 to 0.01)
NDVI, SD, 500 m	−0.04 (−0.07 to −0.01)	−0.02 (−0.06 to 0.03)	−0.03 (−0.06 to 0.00)
Distance to green space (CORINE), m	0.00 (−0.03 to 0.02)	0.05 (0.01 to 0.08)	0.02 (−0.01 to 0.06)
Socioeconomic disadvantage			
Area-level socioeconomic index	−0.02 (−0.06 to 0.03)	0.01 (−0.02 to 0.03)	−0.02 (−0.06 to 0.01)

Abbreviations: BAMSE, Children, Allergy, Millieu, Stockholm, Epidemiology study; CORINE, Copernicus Land Monitoring Service Land Cover; ELSPAC-CZ, Czech European Longitudinal Study of Pregnancy and Childhood; MSAVI, Modified Soil Adjusted Vegetation Index; NDVI, Normalized Difference Vegetation Index; NO_2_, nitrogen dioxide; O_3_, ozone; PIAMA, Prevention and Incidence of Asthma and Mite Allergy; PM_10_, particulate matter with diameter <10 µg/m^3^; PM_2.5_, particulate matter with diameter <2.5 µg/m^3^; RAD, radiance.

^a^ Coefficient estimates are reported for changes in exposure IQR units with 95% confidence intervals from the single pollutant models.

## Discussion

This multicountry cohort study provides several insights into the impacts of changes in the external exposome (air pollution, built environment, and socioeconomic disadvantage) on changes in youth z-BMI trajectories. First, the most consistent association was found for the built environment, where relocation to areas with more impervious surfaces and less green or a greater distance to green space was associated with a subsequent z-BMI increase in all 3 cohorts. The findings for air pollution and socioeconomic disadvantage were less consistent across cohorts. Second, the final models indicated independent associations between each domain of the external exposome and z-BMI change, with minimal confounding with the other domains. Third, there was some heterogeneity in effect modification by age, sex, and parental education between 3 external exposome domains and z-BMI trajectories across countries.

Several biological and social mechanisms may play a role in our observed associations. The biological mechanisms linking air pollution and body weight may be mediated through physiological stress response^38^ or changes in daily activities, such as fewer outdoor activities due to traffic. Associations between changes in the built environment and z-BMI may be explained by physiological stress,^39,40^ as well as possible changes in health behaviors including transport mode,^41,42^ leisure physical activity,^43^ and dietary habits.^44^ Changes in health behaviors following relocation are context dependent and are influenced by individual, cultural, and geographical characteristics.^45,46^ For example, the association of relocation on weight gain may depend on level of urbanization.^47^ This complex interplay between individual, social, and geographical characteristics and the behavioral and health response to changes in the living environment could explain the heterogeneity in the magnitude and direction of associations across cohorts in our findings.

Reported associations between air pollution and weight gain are inconsistent,^9^ in line with our results that identified differences in the direction of associations across cohorts. Associations between increasing exposome hazards and z-BMI increase in PIAMA and children with low parental education in ELSPAC-CZ are consistent with a previous study in Catalan children^48^ and another reporting associations between early-life exposure to air pollution and built environment and BMI.^14^ The reason for the protective association of moving to more polluted areas and reduced z-BMI in BAMSE is not clear but could be driven by differences in relocation preferences and individual interactions with the external exposome across regions, highlighted by opposite effect modification by parental education in BAMSE compared with the other cohorts.

We observed an association of moving to greater socioeconomic disadvantage and z-BMI increase in PIAMA but not in the other cohorts. The PIAMA results are in line with the Moving to Opportunity study,^49^ which systematically randomized residential relocation and demonstrated a lower risk of obesity among people relocating to less deprived neighborhoods, with positive long-term outcomes (eg, high school attendance) in early childhood.^50^ Despite extensive evidence on the role of socioeconomic disparities in overweight^18,51,52,53^ and other cardiometabolic health outcomes,^50^ only one cohort in our analysis reported a clear association between socioeconomic disadvantage and weight. Differences across cohorts could be explained by different socioeconomic indicators, as well as the limited within-individual changes in area-level socioeconomic status following relocation in BAMSE and ELSPAC-CZ. Differential family, cultural, and socioeconomic backgrounds may also affect the health behavior response among relocating children.^54^ Finally, changes in neighborhood SEP investigated in our study may be less relevant compared with the household SEP in some population groups.

### Strengths and Limitations

Our study presents several strengths and novelties. To our knowledge, this is the first study using residential relocation as a quasi-experimental design to investigate the impacts of the living environment on health in an exposome framework, since most “movers” studies solely focused on one or a small group of exposures.^20^ Residential relocation can cause simultaneous changes in multiple environmental exposures and hence provide a unique opportunity to estimate the health impacts that follow sudden changes in the external exposome. Few studies have included socioeconomic disadvantage as a central part of the external exposome rather than a confounder.^55^ With our approach based on precise individual, harmonized exposure estimates, we could estimate the individual and combined impact of different external exposome domains on z-BMI across more than 4000 children from 3 European countries. Relocation studies are particularly useful to triangulate evidence and establish relationships in observational studies by estimating associations based on within-individual changes, limiting the risk of bias from time-invariant confounders.

This study has several limitations. First, exposure assessment was based on residential address history, without accounting for exposures at school or other locations. Second, although our movers-only design reduces bias from baseline differences between individuals, residual confounding due to differential exposure changes associated with life changes (eg, changes in occupation, income, marital status or lifestyle of the parents) cannot be completely ruled out, and time-varying factors associated with relocation may still influence both exposure and outcome. Third, as with any cohort study, loss to follow-up is an important limitation. While follow-up was generally high in early childhood and our analysis was restricted to the first residential move to reduce attrition-related bias, selective participation toward families with higher socioeconomic positions and differential attrition may affect external validity. Moving can be a significant and stressful event that impacts health and behaviors.^56^ This study, like others, lacks data on moving motivations, which might indicate changes in individual disadvantage levels that could not be accounted for.^20^ Because the analyses relied on within-individual variability in exposure and outcome, this approach may have limited statistical power, especially when changes in the external exposome upon moving are modest. Finally, we did not collect information on physical activity, distance to school, or the food environment, which could inform mechanisms underlying changes in the external exposome and weight gain.

## Conclusions

Our findings suggest that sudden changes in the built environment were associated with altered z-BMI trajectories in children and young adults. Promoting healthy living environments may play an important role in maintaining healthy BMI, with potential health benefits over the life course. In contrast, the influence of air pollution and the social environment on z-BMI differed across cohorts, indicating that these associations may depend on the broader context, such as country, setting, or population subgroup.

## Notes

Supplement 1.
eMethods.
**eTable.** Estimates of the association between external exposome domains and z-BMI, stratified by sex, highest parental education, and age at moving **eFigure 1.** Overview of the exposure distributions at pre- and post-move home addresses by cohort **eFigure 2.** Distribution of the change in individual exposures upon moving for all children from the three included cohorts **eFigure 3.** Overview of the contribution of the selected environmental exposure to the urban exposome clusters, by domain in BAMSE (left), PIAMA (middle), and ELSPAC-CZ (right) **eFigure 4.** Visualization of the correlation between the air pollution clusters and individual exposure variables in BAMSE (left), PIAMA (middle) and ELSPAC-CZ (right panel) **eFigure 5.** Visualization of the correlation between the built environment clusters and individual exposure variables in BAMSE **eFigure 6.** Visualization of the correlation between the built environment clusters and individual exposure variables in PIAMA **eFigure 7.** Visualization of the correlation between the built environment clusters and individual exposure variables in ELSPAC-CZ **eFigure 8.** Changes in cluster levels for three domains of the external exposome upon moving
eReferences

Supplement 2.
Data Sharing Statement
