# Supplementary material for: Weight Trajectories Among Youths Following Residential Relocation
Source: JAMA Netw Open. 2025 Nov 18;8(11):e2544164. doi: 10.1001/jamanetworkopen.2025.44164 (PMC12628102; doi:10.1001/jamanetworkopen.2025.44164)
Supplement: Supplement 1. — eMethods. eTable. Estimates of the association between external exposome domains and z-BMI, stratified by sex, highest parental education, and age at moving eFigure 1. Overview of the exposure distributions at pre- and post-move home addresses by cohort eFigure 2. Distribution of the change in individual exposures upon moving for all children from the three included cohorts eFigure 3. Overview of the contribution of the selected environmental exposure to the urban exposome clusters, by domain in BAMSE (left), PIAMA (middle), and ELSPAC-CZ (right) eFigure 4. Visualization of the correlation between the air pollution clusters and individual exposure variables in BAMSE (left), PIAMA (middle) and ELSPAC-CZ (right panel) eFigure 5. Visualization of the correlation between the built environment clusters and individual exposure variables in BAMSE eFigure 6. Visualization of the correlation between the built environment clusters and individual exposure variables in PIAMA eFigure 7. Visualization of the correlation between the built environment clusters and individual exposure variables in ELSPAC-CZ eFigure 8. Changes in cluster levels for three domains of the external exposome upon moving eReferences [file jamanetwopen-e2544164-s001.pdf]

Supplemental Online Content

Saucy A, Warkentin S, Milà C, et al. Weight trajectories among youth following residential relocation. *JAMA Netw Open*. 2025;8(11):e2544164. doi:10.1001/jamanetworkopen.2025.44164

eMethods
eTable. Estimates of the association between external exposome domains and z-BMI, stratified by sex, highest parental education, and age at moving
eFigure 1. Overview of the exposure distributions at pre- and post-move home addresses by cohort
eFigure 2. Distribution of the change in individual exposures upon moving for all children from the three included cohorts
eFigure 3. Overview of the contribution of the selected environmental exposure to the urban exposome clusters, by domain in BAMSE (left), PIAMA (middle), and ELSPAC-CZ (right)
eFigure 4. Visualization of the correlation between the air pollution clusters and individual exposure variables in BAMSE (left), PIAMA (middle) and ELSPAC-CZ (right panel)
eFigure 5. Visualization of the correlation between the built environment clusters and individual exposure variables in BAMSE
eFigure 6. Visualization of the correlation between the built environment clusters and individual exposure variables in PIAMA
eFigure 7. Visualization of the correlation between the built environment clusters and individual exposure variables in ELSPAC-CZ
eFigure 8. Changes in cluster levels for three domains of the external exposome upon moving
eReferences

This supplemental material has been provided by the authors to give readers additional information about their work.

© 2025 Saucy A et al. JAMA Network Open.

1. **eMethods**

Graphical overview of the study population and design:
We included all BMI data for the first two addresses after the age of 2. Changes in three domains of the urban exposome were evaluated at the time of moving.

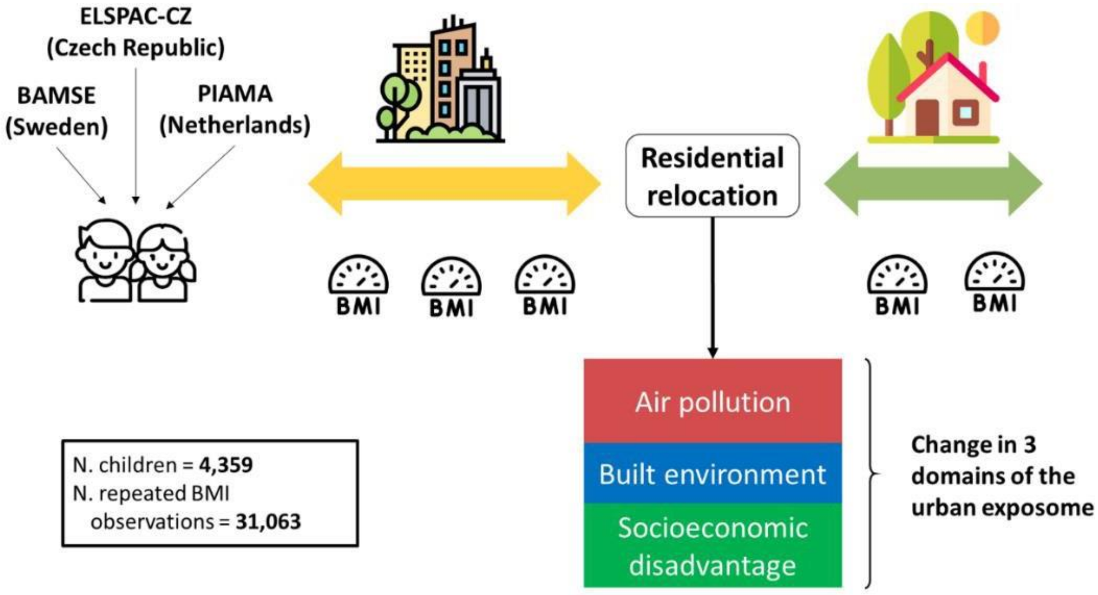

Characterizing the external exposome:
High-resolution ambient air pollution surfaces were developed specifically for the EXPANSE project using geographically and temporally weighted regression built for each pollutant from 2000 to 2019 (Shen et al., 2022). In ELSPAC-CZ, Air pollution surfaces scaled back in 2000 were adjusted based on estimated concentrations from the Danish Eulerian Hemispheric Model (DEHM) (Brandt et al., 2012). Residential surrounding greenness was assessed using satellite-derived NDVI (Normalized Difference Vegetation Index) and mSAVI (Modified Soil Adjusted Vegetation Index) imagery data derived from the Vegetation Indices (MOD13Q1) product of the Terra Moderate Resolution Imaging Spectroradiometer (MODIS) with 250 m × 250 m resolution (Didan, 2015). Accessibility to green spaces was defined as the distance to the nearest green space using the Corine Land Cover (European Environment Agency, 2012) and Urban Atlas (Copernicus Land Monitoring Services, 2020). Distance to the nearest blue space was assessed using the EU-Hydro map developed by the Copernicus Land Monitoring Service

© 2025 Saucy A et al. JAMA Network Open.

(Copernicus Land Monitoring Service, 2019). Grey (i.e. built-up) spaces were characterized using imperviousness density maps (Status Maps — Copernicus Land Monitoring Service, 2020). Light-at-night was assessed using harmonized data produced with DMSP (Defense Meteorological Satellite Program) and VIIRS (Version 1 VIIRS Day/Night Band Night-time Lights) data sources (Elvidge et al., 2017).

Study flow diagram
"N" refers to the individuals, "obs" refers to the individual z-BMI observations.

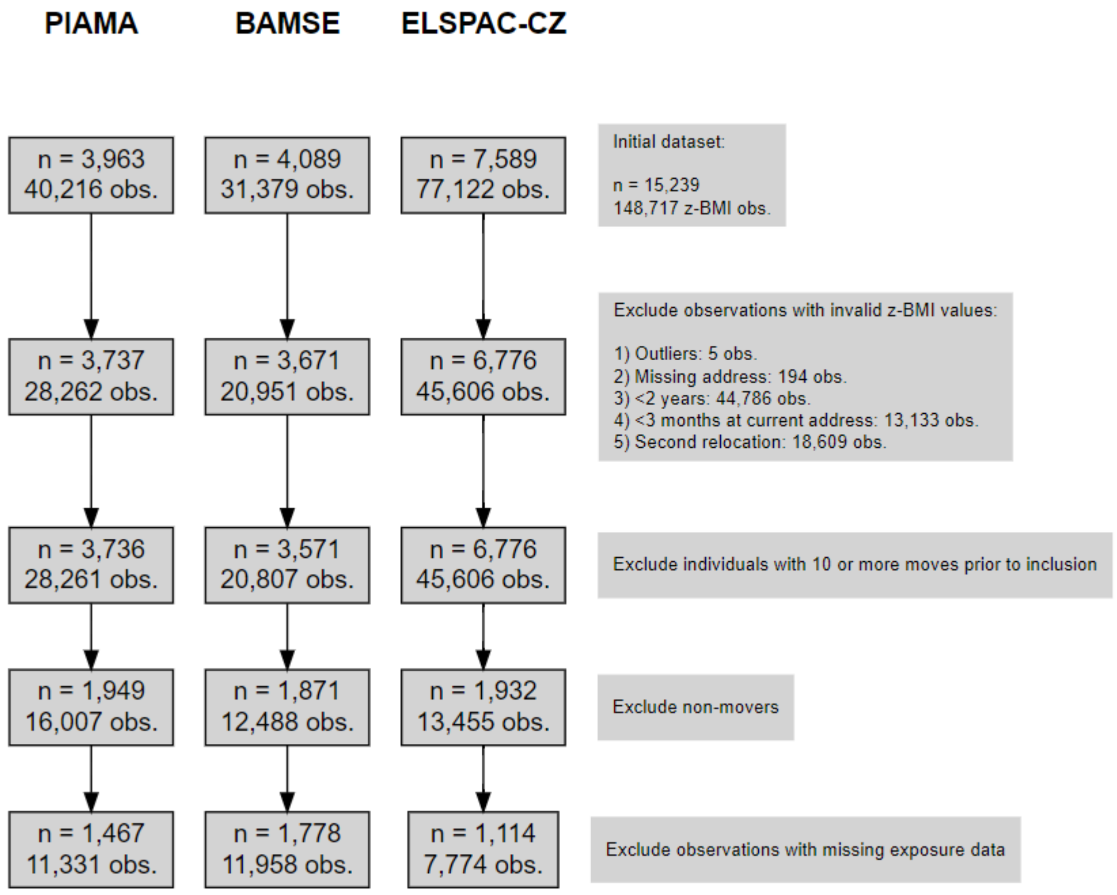

© 2025 Saucy A et al. JAMA Network Open.

2. Supplementary Tables

eTable 1: Estimates of the association between external exposome domains and z-BMI, stratified by sex, highest parental education, and age at moving. Results from the mutually adjusted models (main models), for each cohort separately. Exponentiated coefficients are displayed with 95% confidence intervals. Significant associations at level α=5% are highlighted in bold.

Domain		BAMSE		ELSPAC-CZ		PIAMA	
	**Hazard cluster (ref. = "Low")**	**Medium**	**High**	**Medium**	**High**	**Medium**	**High**
**Built Environment**	**Main model**	-0.06 [-0.13: 0.01]	**0.13 [0.04: 0.23]**	-0.12 [-0.33: 0.08]	0.05 [-0.21: 0.30]	0.01 [-0.08: 0.10]	0.04 [-0.07: 0.14]
	**Sex at birth**						
	Girls	-0.03 [-0.10: 0.04]	0.03 [-0.07: 0.13]	0.10 [-0.05: 0.25]	0.03 [-0.16: 0.22]	0.09 [-0.01: 0.18]	0.14 [0.03: 0.25]
	Boys	-0.02 [-0.10: 0.05]	**0.23 [0.12: 0.34]**	**-0.20 [-0.37: -0.03]**	-0.08 [-0.28: 0.12]	0.00 [-0.10: 0.10]	-0.05 [-0.17: 0.07]
	**Parental Education**						
	Low	**-0.63 [-1.03: -0.22]**	0.56 [-0.16: 1.27]	**2.80 [0.29: 5.30]**	**3.26 [0.55: 5.97]**	**0.42 [0.11: 0.73]**	**0.36 [0.06: 0.67]**
	Medium	0.05 [-0.04: 0.13]	0.11 [-0.01: 0.23]	-0.17 [-0.34: 0.01]	-0.19 [-0.41: 0.03]	0.02 [-0.11: 0.14]	-0.03 [-0.18: 0.11]
	High	-0.06 [-0.13: 0.01]	**0.13 [0.04: 0.23]**	-0.12 [-0.33: 0.08]	0.05 [-0.21: 0.30]	0.01 [-0.08: 0.10]	0.04 [-0.07: 0.14]
	**Age at moving (years)**						
	≤7	0.00 [-0.07: 0.06]	**0.18 [0.08: 0.28]**	-0.09 [-0.26: 0.09]	0.04 [-0.18: 0.25]	0.02 [-0.08: 0.12]	-0.03 [-0.15: 0.09]
	>7	-0.06 [-0.15: 0.02]	0.07 [-0.04: 0.19]	0.01 [-0.13: 0.16]	-0.01 [-0.19: 0.17]	0.05 [-0.05: 0.16]	0.11 [-0.01: 0.23]
**Socioeconomic Disadvantage**	**Main model**	0.04 [-0.02: 0.10]	-0.02 [-0.15: 0.10]	0.03 [-0.04: 0.10]	-0.06 [-0.18: 0.05]	0.02 [-0.05: 0.08]	0.06 [-0.02: 0.14]
	**Sex at birth**						
	Girls	**0.14 [0.05: 0.22]**	0.07 [-0.09: 0.24]	0.05 [-0.05: 0.14]	-0.03 [-0.19: 0.12]	0.02 [-0.06: 0.11]	0.02 [-0.08: 0.12]
	Boys	-0.04 [-0.13: 0.05]	-0.15 [-0.34: 0.04]	0.02 [-0.08: 0.13]	-0.06 [-0.22: 0.10]	0.02 [-0.07: 0.12]	**0.13 [0.01: 0.25]**
	**Parental Education**						
	Low	0.31 [-0.57: 1.18]	-0.14 [-1.45: 1.17]	0.56 [-0.14: 1.27]	-0.29 [-1.06: 0.49]	-0.01 [-0.29: 0.28]	-0.23 [-0.53: 0.07]
	Medium	**0.11 [0.01: 0.21]**	0.17 [-0.03: 0.38]	0.09 [-0.03: 0.20]	0.10 [-0.07: 0.27]	-0.02 [-0.14: 0.09]	-0.07 [-0.22: 0.07]
	High	0.00 [-0.08: 0.08]	-0.12 [-0.27: 0.04]	0.10 [-0.03: 0.23]	-0.03 [-0.32: 0.25]	0.02 [-0.05: 0.10]	**0.18 [0.08: 0.28]**

© 2025 Saucy A et al. JAMA Network Open.

	**Age at moving (years)**						
	≤7	0.00 [-0.09: 0.09]	-0.04 [-0.21: 0.14]	0.00 [-0.11: 0.11]	-0.06 [-0.24: 0.11]	0.01 [-0.08: 0.11]	-0.02 [-0.13: 0.09]
	>7	0.08 [-0.01: 0.17]	-0.04 [-0.22: 0.14]	0.05 [-0.04: 0.15]	-0.06 [-0.21: 0.08]	0.00 [-0.08: 0.09]	**0.16 [0.05: 0.27]**
**Air pollution**	**Main model**	-0.08 [-0.16: 0.00]	**-0.13 [-0.22: -0.03]**	0.05 [-0.03: 0.13]	-0.05 [-0.30: 0.19]	0.08 [-0.01: 0.17]	**0.12 [0.01: 0.23]**
	**Sex at birth**						
	Girls	-0.06 [-0.16: 0.05]	-0.07 [-0.19: 0.06]	0.08 [-0.03: 0.19]	-0.11 [-0.39: 0.17]	0.06 [-0.06: 0.17]	0.04 [-0.11: 0.19]
	Boys	-0.08 [-0.20: 0.03]	**-0.18 [-0.31: -0.04]**	0.03 [-0.09: 0.14]	0.14 [-0.32: 0.60]	0.08 [-0.06: 0.22]	**0.18 [0.01: 0.34]**
	**Parental Education**						
	Low	-0.20 [-0.83: 0.43]	0.04 [-0.71: 0.79]	-0.19 [-1.01: 0.64]	1.13 [-1.15: 3.41]	-0.13 [-0.52: 0.25]	-0.19 [-0.68: 0.30]
	Medium	-0.07 [-0.19: 0.05]	-0.12 [-0.26: 0.02]	0.08 [-0.05: 0.22]	**-0.52 [-0.94: -0.10]**	0.07 [-0.09: 0.23]	0.08 [-0.14: 0.29]
	High	-0.09 [-0.20: 0.02]	**-0.14 [-0.27: -0.02]**	-0.08 [-0.23: 0.07]	0.10 [-0.32: 0.52]	0.08 [-0.03: 0.19]	**0.15 [0.01: 0.28]**
	**Age at moving (years)**						
	≤7	**-0.14 [-0.27: -0.01]**	**-0.25 [-0.39: -0.10]**	0.03 [-0.08: 0.13]	0.01 [-1.12: 1.14]	0.10 [-0.02: 0.22]	**0.17 [0.03: 0.32]**
	>7	-0.04 [-0.14: 0.05]	0.00 [-0.14: 0.13]	0.06 [-0.06: 0.18]	-0.04 [-0.31: 0.22]	0.04 [-0.10: 0.18]	0.06 [-0.12: 0.23]

© 2025 Saucy A et al. JAMA Network Open.

3. Supplementary Figures

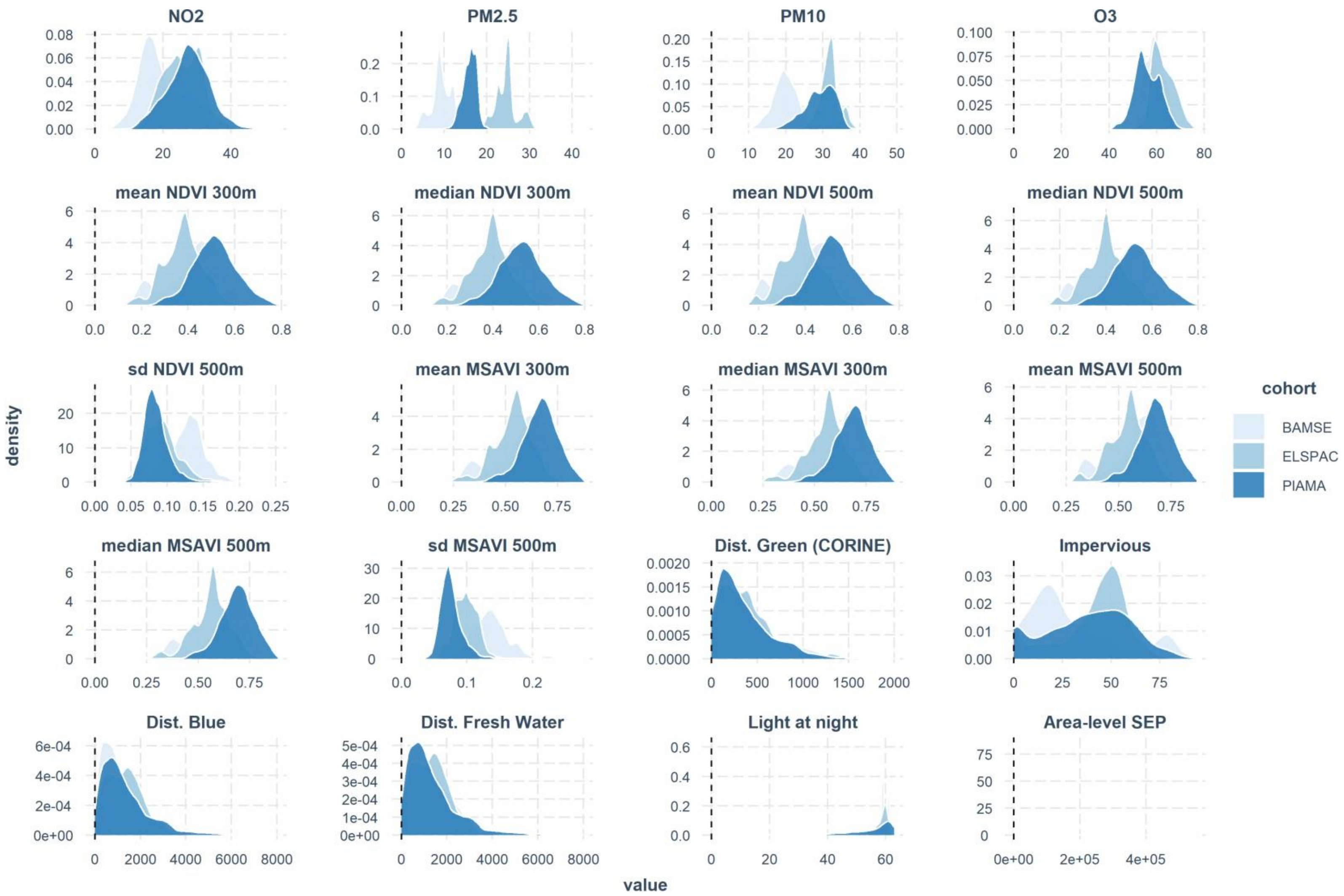

eFigure 1: Overview of the exposure distributions at pre- and post-move home addresses by cohort.

© 2025 Saucy A et al. JAMA Network Open.

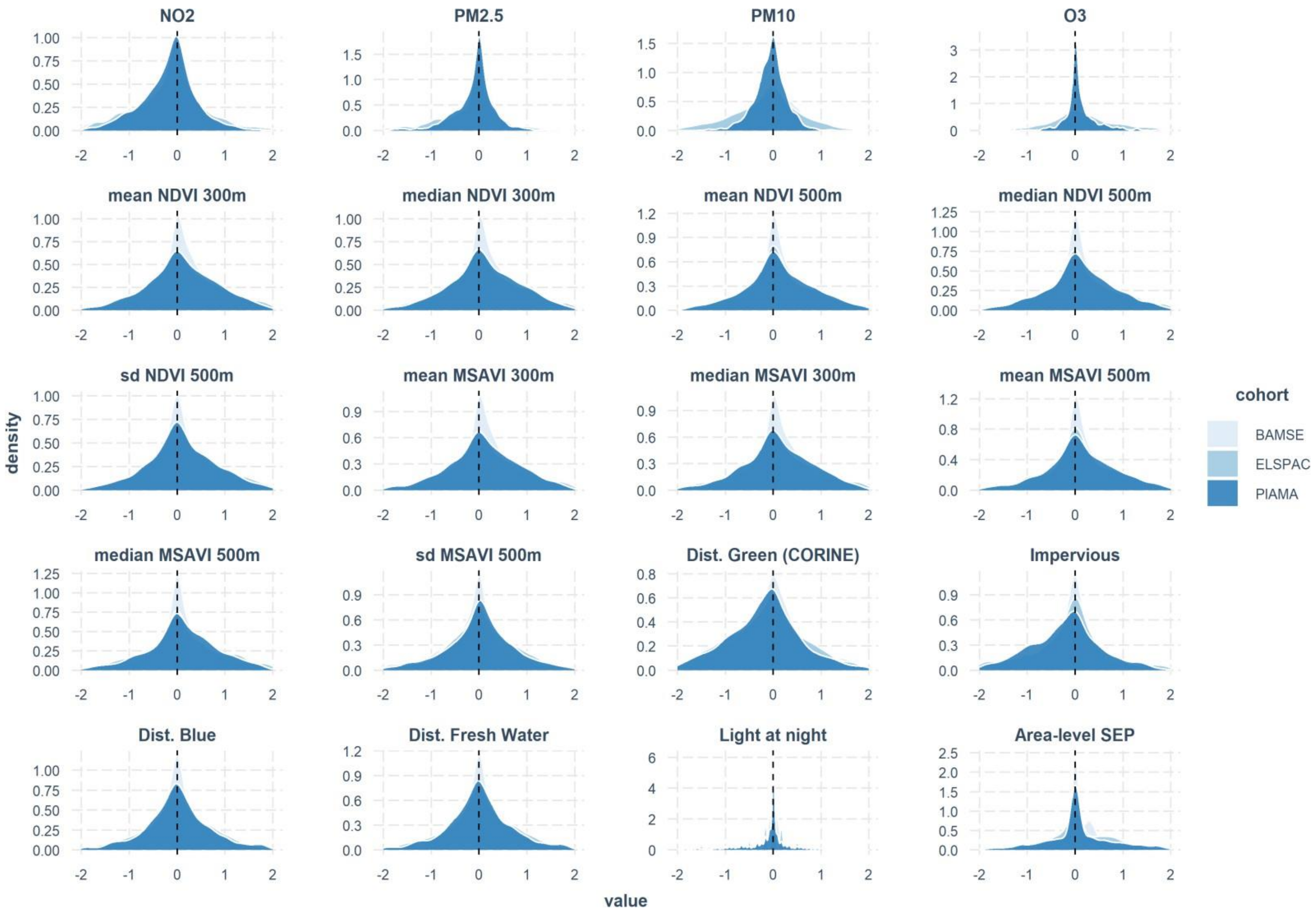

eFigure 2: Distribution of the change in individual exposures upon moving for all children from the three included cohorts.

© 2025 Saucy A et al. JAMA Network Open.

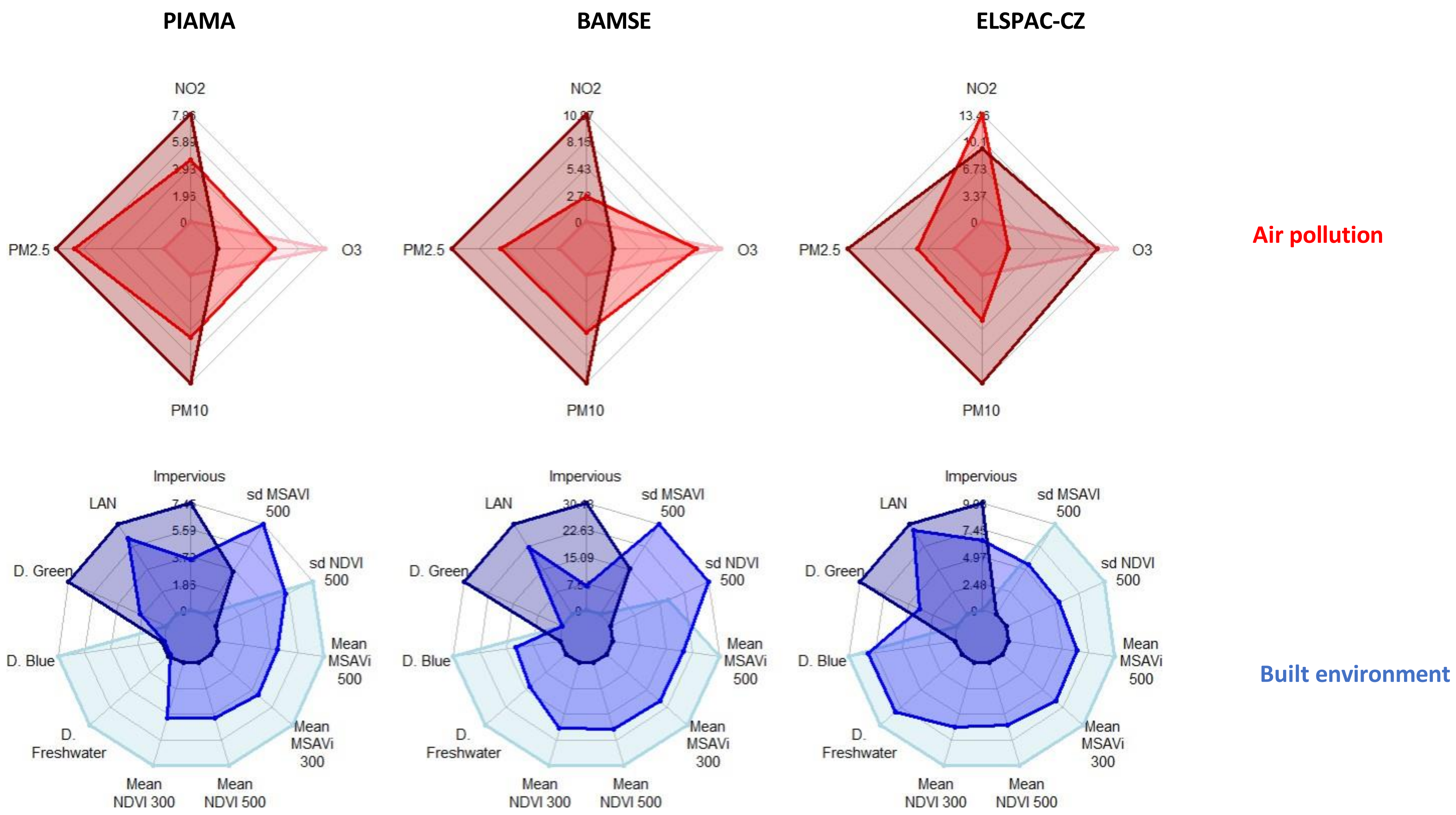

eFigure 3: Overview of the contribution of the selected environmental exposure to the urban exposome clusters, by domain in BAMSE (left), PIAMA (middle), and ELSPAC-CZ (right). Points located towards the center of the spider webs indicate low contribution of the exposure of interest to the clusters. Points located towards the edges of the web indicate high contributions. The highest air pollution cluster level is dispalyed in darker shade of red (top panel) and contains higher concentrations of NO_2 and particulate matter ($PM_{2.5}$ and PM_{10}), and lower ozone concentrations (O_3). The highest built environment cluster group (bottom panel) is displayed in dark blue and contains higher values of imperviousness and light at night, and lower greenness (NDVI, mSAVI, distance to green space).

© 2025 Saucy A et al. JAMA Network Open.

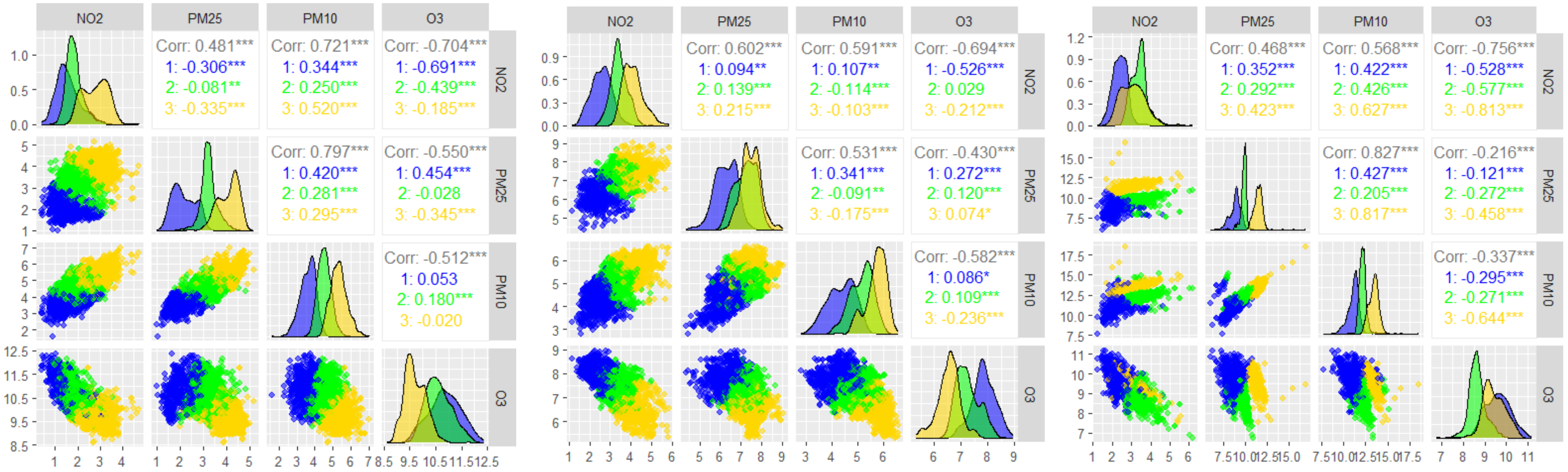

eFigure 4: Visualization of the correlation between the air pollution clusters and individual exposure variables in BAMSE (left), PIAMA (middle) and ELSPAC-CZ (right panel). Low cluster values are displayed in blue, medium in green and high in yellow.

© 2025 Saucy A et al. JAMA Network Open.

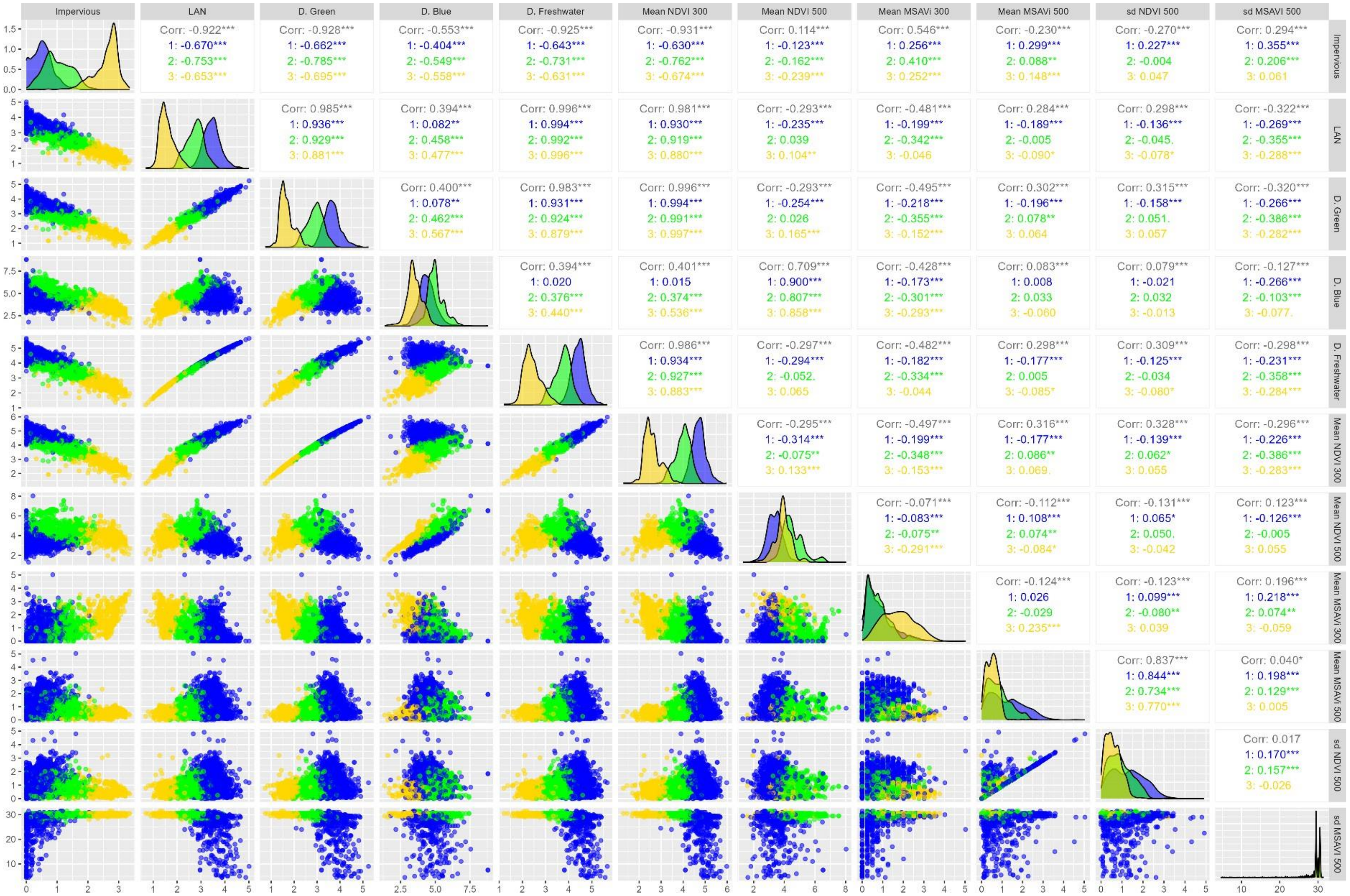

eFigure 5: Visualization of the correlation between the built environment clusters and individual exposure variables in BAMSE. Low cluster values are displayed in blue, medium in green and high in yellow.

© 2025 Saucy A et al. JAMA Network Open.

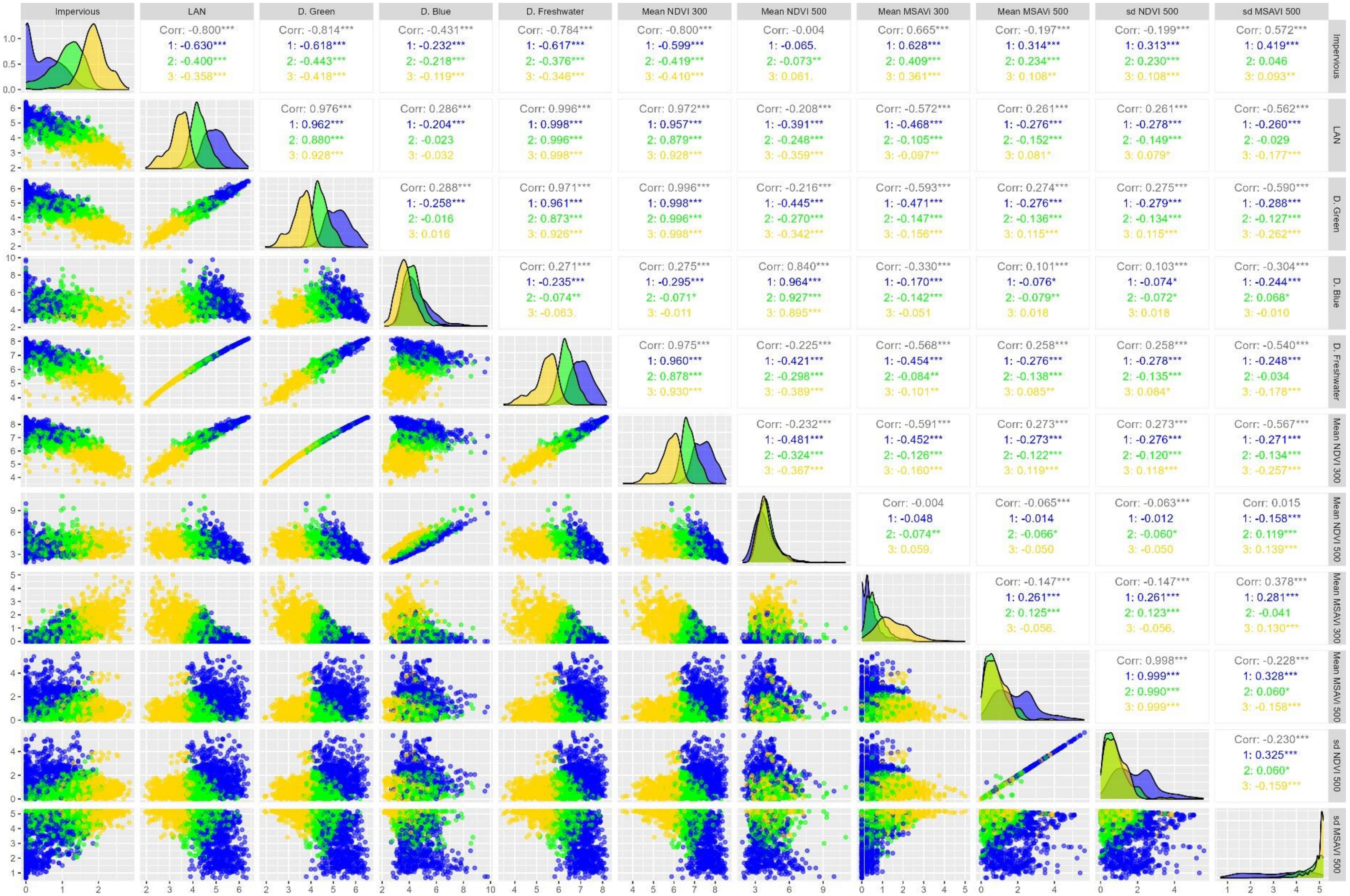

eFigure 6: Visualization of the correlation between the built environment clusters and individual exposure variables in PIAMA. Low cluster values are displayed in blue, medium in green and high in yellow.

© 2025 Saucy A et al. JAMA Network Open.

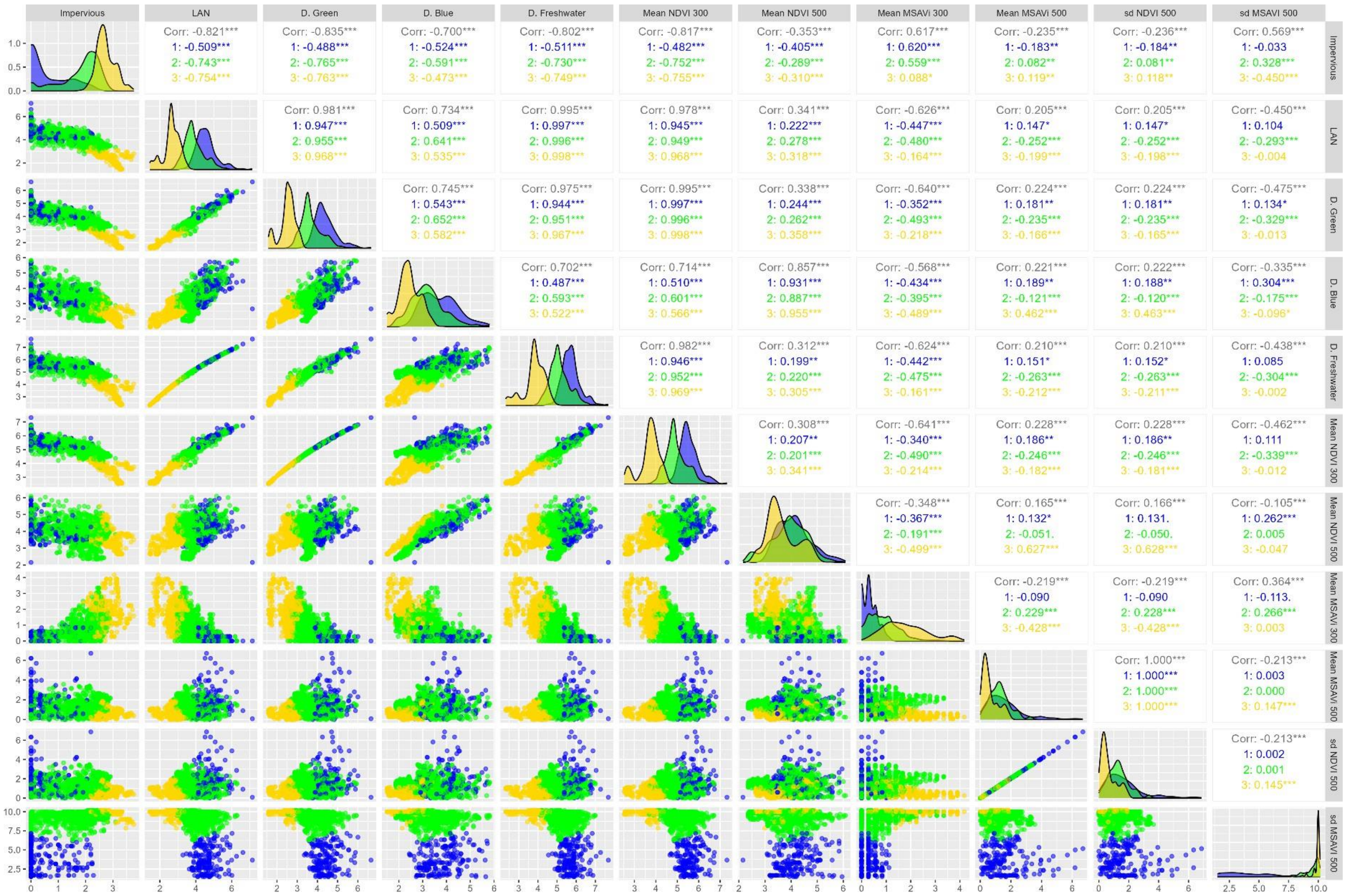

eFigure 7: Visualization of the correlation between the built environment clusters and individual exposure variables in ELSPAC-CZ. Low cluster values are displayed in blue, medium in green and high in yellow.

© 2025 Saucy A et al. JAMA Network Open.

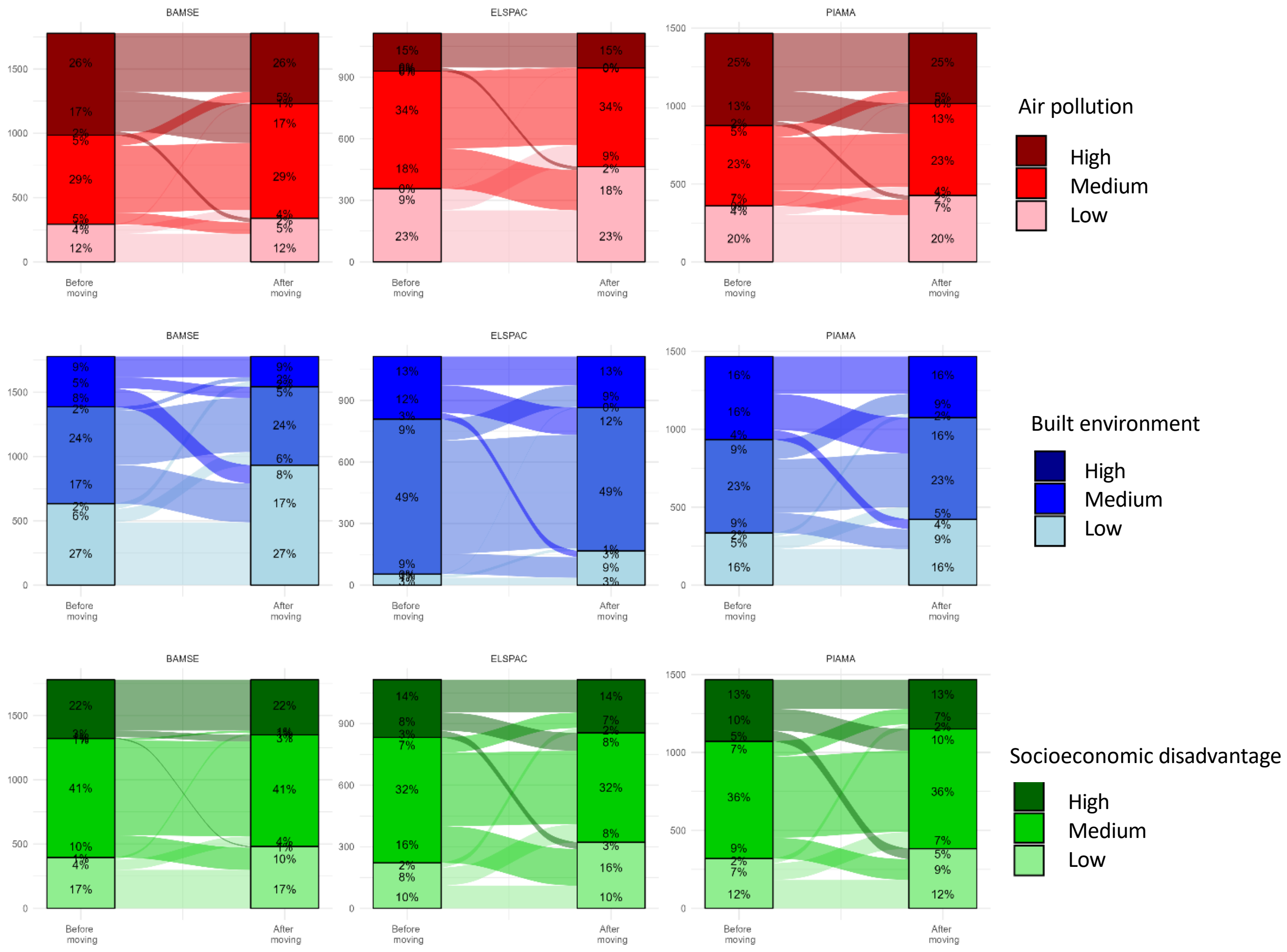

eFigure 8: Changes in cluster levels for three domains of the external exposome upon moving. Lower hazard cluster levels represent lower levels of air pollution, built environment and socioeconomic disadvantage. Numbers indicate the percentage of study participants in the different clusters trajectories upon moving (e.g. in BAMSE, 26% were and stayed in the high hazard cluster, 17% moved from the high to the medium hazard cluster, and 2% moved from the high to the low hazard cluster). Note: clusters were built separately for each cohort and cluster distributions at given times cannot be compared across cohorts.

© 2025 Saucy A et al. JAMA Network Open.

eReferences

Brandt, J., Silver, J. D., Frohn, L. M., Geels, C., Gross, A., Hansen, A. B., Hansen, K. M., Hedegaard, G. B., Skjøth, C. A., & Villadsen, H. (2012). An integrated model study for Europe and North America using the Danish Eulerian Hemispheric Model with focus on intercontinental transport of air pollution. *Atmospheric Environment*, *53*, 156–176.

Copernicus Land Monitoring Service. (2019). https://land.copernicus.eu/imagery-in-situ/eu-hydro

Copernicus Land Monitoring Services. (2020). *Urban Attas*.

Didan, K. (2015). *MOD13Q1 MODIS/Terra Vegetation Indices 16-Day L3 Global 250m SIN V006 (Data set)*.

Elvidge, C. D., Baugh, K., Zhizhin, M., Hsu, F. C., & Ghosh, T. (2017). VIIRS night-time lights. *International Journal of Remote Sensing*, *38*(21), 5860–5879.

European Environment Agency. (2012). *Corine Land Cover (CLC) 2012.*

Shen, Y., de Hoogh, K., Schmitz, O., Clinton, N., Tuxen-Bettman, K., Brandt, J., Christensen, J. H., Frohn, L. M., Geels, C., Karssenberg, D., Vermeulen, R., & Hoek, G. (2022). Europe-wide air pollution modeling from 2000 to 2019 using geographically weighted regression. *Environment International*, *168*(May). https://doi.org/10.1016/j.envint.2022.107485

Status Maps — Copernicus Land Monitoring Service. (2020). https://land.copernicus.eu/pan-european/high-resolution-layers/imperviousness/status-maps

© 2025 Saucy A et al. JAMA Network Open.
