# Supplementary material for: Weight Trajectories Among Youths Following Residential Relocation
Source: JAMA Netw Open. 2025 Nov 18;8(11):e2544164. doi: 10.1001/jamanetworkopen.2025.44164 (PMC12628102; doi:10.1001/jamanetworkopen.2025.44164)
Supplement: Supplement 2. — Data Sharing Statement [file jamanetwopen-e2544164-s002.pdf]

Data Sharing Statement

Saucy. Weight Trajectories Among Youth Following Residential Relocation. *JAMA Netw Open*. Published November 18, 2025. doi:10.1001/jamanetworkopen.2025.44164

Data
Data available: No

Additional Information
Explanation for why data not available: Code and anonymized sample of the data can be made available upon request. The individual data cannot be shared on a public repository because of data protection.
